# Silver nanoparticle with potential antimicrobial and antibiofilm efficiency against multiple drug resistant, extensive drug resistant *Pseudomonas aeruginosa* clinical isolates

**DOI:** 10.1186/s12866-024-03397-z

**Published:** 2024-07-26

**Authors:** Amal M. Abo Kamer, Gamal M. El Maghraby, Maha Mohamed Shafik, Lamiaa A. Al-Madboly

**Affiliations:** 1 Department of Pharmaceutical Microbiology, Faculty of Pharmacy, Tanta University, Gharbia government, El Geish street, Tanta, Egypt; 2 Department of Pharmaceutical Technology, Faculty of Pharmacy, Tanta University, Tanta, Egypt

**Keywords:** Nanoparticles, Microbial resistance, Biofilm formation, Silver nitrate, Cephalosporins, Fluoroquinolones, Aminoglycosides

## Abstract

**Background:**

The study aims to investigate the effect of combining silver nanoparticles (AGNPs) with different antibiotics on multi-drug resistant (MDR) and extensively drug resistant (XDR) isolates of *Pseudomonas aeruginosa* (*P. aeruginosa*) and to investigate the mechanism of action of AGNPs.

**Methods:**

AGNPs were prepared by reduction of silver nitrate using trisodium citrate and were characterized by transmission electron microscope (TEM) in addition to an assessment of cytotoxicity. Clinical isolates of *P. aeruginosa* were collected, and antimicrobial susceptibility was conducted. Multiple Antibiotic Resistance (MAR) index was calculated, and bacteria were categorized as MDR or XDR. Minimum inhibitory concentration (MIC) of gentamicin, ciprofloxacin, ceftazidime, and AGNPs were determined. The mechanism of action of AGNPs was researched by evaluating their effect on biofilm formation, swarming motility, protease, gelatinase, and pyocyanin production. Real-time PCR was performed to investigate the effect on the expression of genes encoding various virulence factors.

**Results:**

TEM revealed the spherical shape of AGNPs with an average particle size of 10.84 *±* 4.64 nm. AGNPS were safe, as indicated by IC50 (42.5 µg /ml). The greatest incidence of resistance was shown against ciprofloxacin which accounted for 43% of the bacterial isolates. Heterogonous resistance patterns were shown in 63 isolates out of the tested 107. The MAR indices ranged from 0.077 to 0.84. Out of 63 *P. aeruginosa* isolates, 12 and 13 were MDR and XDR, respectively. The MIC values of AGNPs ranged from 2.65 to 21.25 µg /ml. Combination of AGNPs with antibiotics reduced their MIC by 5–9, 2–9, and 3-10Fold in the case of gentamicin, ceftazidime, and ciprofloxacin, respectively, with synergism being evident. AGNPs produced significant inhibition of biofilm formation and decreased swarming motility, protease, gelatinase and pyocyanin production. PCR confirmed the finding, as shown by decreased expression of genes encoding various virulence factors.

**Conclusion:**

AGNPs augment gentamicin, ceftazidime, and ciprofloxacin against MDR and XDR *Pseudomonas* isolates. The efficacy of AGNPs can be attributed to their effect on the virulence factors of *P. aeruginosa*. The combination of AGNPs with antibiotics is a promising strategy to attack resistant isolates of *P. aeruginosa*.

## Background

*Pseudomonas aeruginosa* is one of the most virulent microorganisms causing many medical conditions. The effect of *Pseudomonas* is generally aggravated in immunocompromised patients [1]. The organism is categorized as one of the most frequently hospital-acquired pathogens, which may result in the urinary tract, respiratory, soft tissue, bone, joint, and gastrointestinal infections, dermatitis, bacteremia, and various systemic infections [2]. Recurrent infection with *Pseudomonas* resulted in the development of resistant isolates to various antibiotic classes [3]. The pathogenicity and resistance of *P. aeruginosa* are related to many factors. For example, *P. aeruginosa* cells communicate via quorum sensing process, which is believed to be critical in bacterial pathogenesis [4]. *P. aeruginosa* can develop alternative virulence factors such as motility, phenazines, alginate, proteases, phospholipase C, rhamnolipid, pili, and pyocyanin. These factors with biofilm formation are the major factors contributing to the development of resistance against different antibiotic classes [5]. The problem of resistance requires the development of new chemical entities, but this requires effort, time, and high cost. Accordingly, authors started trying combined therapy and manipulation of advanced drug delivery strategies to improve the specification and widen the spectrum of existing antibiotics. Recently, great interest has been directed to the application of nanoparticulate carriers for this purpose, with promising results being published [6]. Research studies were also directed to apply metal nanoparticles as antibacterial agents, which can be adopted alone or in combination with existing antibiotics. For example, metal oxide nanoparticles, such as silver and zinc oxide nanoparticles, showed promising efficacy against resistant strains [7, 8]. The ease of preparation, safety and stability of silver nanoparticles (AGNPs) encouraged researchers to test their efficacy against many organisms [9, 10]. A comparison between silver nanoparticles and other metallic nanoparticles is presented in Table 1 [11–14]. Historically, silver has been employed as an antiseptic and antimicrobial against Gram-positive and Gram-negative bacteria [15]. Examples of applications of AGNPs included the efficacy against *E. coli, Klebsiella pneumoniae, Enterococcus faecalis, Acinetobacter baumannii, Salmonella typhi, and Staphylococcus aureus* [8]. Moreover, combination of sliver nanoparticles with antibiotics provided a chance for using low concentration of silver nanoparticles which can reduce toxic effects [16]. The success of AGNPs was extended to augment the efficacy of other antibiotics, including aztreonam, ampicillin, oxacillin, and tetracycline against *P. aeruginosa* [17, 18], and Vancomycin against *E. coli*, *S. aureus*, *Micrococcus luteus*, and *Acinetobacter baumanii* [19]. Despite the recorded success of AGNPs alone or in combination with other antibiotics in the eradication of resistant microorganisms, no systematic investigation is available on the mechanism of action of AGNPs. Accordingly, the objective of the current study is to investigate the antibacterial activity of AGNPs alone or in combination with selected antibiotics. The selected antibiotics included three different categories (cephalosporins (ceftazidime), aminoglycosides (gentamicin) and quinolones (ciprofloxacin) and the tested organism was MDR and XDR *Pseudomonas* isolates. The goal is to provide systematic investigation of potential effects of silver nanoparticles on the biofilm and virulence factors.

**Table 1:** Comparison between silver and other metallic nanoparticles

Parameters	Silver nanoparticles	Copper and copper oxide nanoparticles	Gold nanoparticles	Zinc oxide nanoparticles
Method of preparation	1. Conventional Chemical synthesis 2. Green synthesis using bacteria, fungi, plant extract. 3. Biosynthesis using usnic acid and thymol related to *P. muralis* lichen and *Artemisia haussknechtii* plant species. 4. Physical Methods using evaporation/condensation, laser ablation. 5. Controlled Thermolysis 6. Biochemical Methods using plants, algae, yeasts, fungi, bacteria and viruses in combination with chemical reagents. 7. Electrochemical Methods 8. Ultrasound assisted reduction of metal precursor.	1. Cementation methods. 2. Chemical Reduction method 3. Green synthesis using bacteria, fungi, plant extract and algae. 4. Physical method using laser ablation. 5. Biosynthesis using usnic acid and thymol related to *P. muralis* lichen and *Artemisia haussknechtii* plant species.	1. Biochemical methods using plants, algae, yeasts, fungi, bacteria and viruses in combination with chemical reagents. 2. Electrochemical methods. 3. Physical methods by the reduction reaction of chloroauric acid followed by controlled agglomeration. 4. Green synthesis using bacteria, fungi, plant extract and algae.	1. Green synthesis using plant extract. 2. The solution-based routes as chemical controlled precipitation, sol-gel method, solvothermal and hydrothermal method, method using an emulsion or microemulsion environment. 3. Chemical synthesis. 4. Biosynthesis by the lichen *Lecanora muralis*
Methods of administration	Oral, Topical, Pulmonary and Intravenous injection.	Oral, Topical, Pulmonary and Intravenous injection.	Dermal, oral, Intraperitoneal and Intravenous injection	Intravenous injection, Oral and Dermal
Antimicrobial activity	Active against *S. aureus, E. coli, K. pneumoniae, P. aeruginosa and methicillin-resistant S. aureus (MRSA), Salmonella typhi, Bacillus subtilis, Vibrio cholerae, E. faecalis, Hafnia alvei, Acinetobacter baumannii, Shigella dysenteriae, Micrococcus Luteus* and *Coliforms* bacteria in water and fecal media.	Active against *S. aureus, E. coli, P. aeruginosa, methicillin resistant S. aureus (MRSA), Salmonella typhi, Bacillus subtilis, Vibrio cholerae, E. faecalis, S. faecalis, S. epidermis, isolate, the spore-forming Bacillus megatherium, B. cereus, P. mirabilis* and *A. caviae*.	Active against *S. aureus, E. coli, P. aeruginosa, S. typhi, Serratia sp, K. pneumoniae, B. subtilis, V. cholerae, E. faecalis, S. typhimurium* and *K. oxytoca*.	Active *S. aureus, E. coli, K. pneumoniae, P. aeruginosa, salmonella sp. Bacteria, S. epidermis, Bacillus subtilis, B. cereus, L. monocytogenes* and *E. faecium.*
Toxicity	Concentration or size dependent: 1. Pulmonary toxicity 2. Dermal toxicity 3. Hepatotoxicity 4. Neurotoxicity	1. Pulmonary toxicity 2. Dermal toxicity 3. Hepatotoxicity 4. Nephrotoxicity 5. Neurotoxicity 6. Cardiotoxicity 7. Genotoxicity	Size dependent: 1. Dermal toxicity 2. Hepatotoxicity 3. Neurotoxicity	1. Pulmonary toxicity 2. Hepatotoxicity 3. Nephrotoxicity 4. Neurotoxicity

## Materials and methods

### Preparation of silver nanoparticles

AGNPs were synthesized according to the well-established method, which employed trisodium citrate (TSC) as a reducing agent (Rashid et al., 2013) [20]. Briefly, silver nitrate was dissolved in distilled water to prepare a solution containing 0.001 M AgNO3. This solution (100 ml) was heated to boil before the dropwise addition of 10 ml of 1% trisodium citrate with continuous mixing while heating. The process continued until the formation of pale-yellow color. The development of this color is taken as an indication of nanoparticle formation. The dispersion was subjected to continuous mixing away from heat until cooling to room temperature. The redox reaction involved in the precipitation of AGNPs is illustrated in the following equation:

$$\eqalign{4A{g^ + } & + {\rm{ }}{C_6}{H_5}{O_7}N{a_3} + {\rm{ }}2{H_2}O{\rm{ }} \to {\rm{ }}4A{g^0} \cr & + {\rm{ }}{C_6}{H_5}{O_7}{H_3} + {\rm{ }}3N{a^ + } + {\rm{ }}{H^ + } + {\rm{ }}{O_2} \uparrow \cr}$$

### Transmission electron microscopy (TEM)

The morphology and size of AGNPs were researched using TEM. This was achieved using JEOL, JEM-2100 electron microscope, Tokyo, Japan. The liquid sample was loaded into a carbon grid before being scanned by an electron microscope.

### Determination of cytotoxicity of silver nanoparticles

The cytotoxicity of AGNPs was determined using MTT assay. A series of concentrations of AGNPs (170, 85, 42.5, 21.25, 10.625, 5.312, 2.65, 1.32, 0.664, 0.332 µg/ml) were prepared. Cytotoxicity was tested on mononuclear cells grown at varying densities (1-5 × 10^6^ cells per ml) in a clear 96-well plate. The tested concentrations of AGNPs were separately added to the wells before 72 h of incubation at 37 ^o^C. The liquid was then carefully aspirated, and 50 µl of serum-free media and 50 µl of MTT reagent were added into each well. The plate was incubated at 37 ^o^C for 3 h. The developed dye was solubilized using 150 µl of MTT solvent. This was achieved by continuous shaking on the orbital shaker for 15 min. The optical density (OD) was measured at 590 nm. The recorded OD was corrected by subtracting the culture medium background and the cytotoxicity was computed using the following equation:

$$\% \,Cytotoxicity = {{(100 \times (OD\,Control - OD\,Sample))} \over {OD\,Control}}$$

### Specimen collection and bacterial strains

Clinical samples (280) were collected from patients admitted to the ENT and surgery departments at Tanta University Hospitals. This involved culturing the samples on cetrimide agar plates; separated bacterial colonies were exposed to conventional identification steps, including Gram staining techniques and biochemical identification, which include indole test negative, KIA test red, and citrate test blue. These investigations confirmed that 107 out of the 280 (61.7%) specimens were *P. aeruginosa*. These isolates were preserved in nutrient broth containing 10% v/v glycerol at – 80 ^ο^C for further studies.

### Antimicrobial susceptibility testing

Antimicrobial susceptibility of all the *P. aeruginosa* isolates was conducted using the Kirby Bauer disc diffusion method. *P. aeruginosa* (ATCC 27,829) was used as reference strain. This was done on Muller Hinton agar (MHA) according to the guidelines of the Clinical Laboratory Standards Institute (CLSI, 2018). The following antibiotics were tested gentamycin (10 µg), tobramycin (10 µg), amikacin (30 µg), imipenem (10 µg), meropenem (10 µg), doripenem (10 µg) ceftazidime (30 µg), cefepime (30 µg), ciprofloxacin (5 µg), levofloxacin (5 µg), piperacillin (100 µg), piperacillin tazobactam (10 µg), aztreonam (30 µg). After 24 h of incubation at 37 ^o^C, the plates were examined and inhibition zone diameters around antibiotic disks were measured. The isolates were categorized as resistant (R), intermediate resistant (I) and sensitive (S) based on the recorded diameter of the inhibition zone according to CLSI, 2018 specifications. The results of this study were employed to compute the multiple antibiotic resistance (MAR) index according to the following equation: [21]

$$\eqalign{ MAR\,index = & \cr & {{{\rm{Number }}\,{\rm{of }}\,{\rm{antibiotics}}\,{\rm{to }}\,{\rm{which }}\,{\rm{the }}\,{\rm{isolate }}\,{\rm{was }}\,{\rm{resistant}}} \over {{\rm{Total}}\,{\rm{number}}\,{\rm{ of }}\,{\rm{antibiotics }}\,{\rm{to }}\,{\rm{which }}\,{\rm{the }}\,{\rm{isolate }}\,{\rm{was }}\,{\rm{subjected}}}} \cr}$$

Determination of multi-drug resistance isolates was done according to Magiorakos et al., (2012) [22], who considered any isolate showing resistance to at least one agent in three or more classes of antimicrobials as multidrug-resistant isolate (MDR). Those showing susceptibility for all agents from one or two antimicrobial classes and recording resistance to at least one agent from other classes is classified as extensively drug resistance (XDR) profile. Pan drug resistance (PDR) is indicated for isolates resistant to all agents in all antimicrobial categories.

### Determining the minimum inhibitory concentration (MIC)

The MIC was determined for gentamicin, ciprofloxacin, ceftazidime, and AGNPs against the 25 resistant *Pseudomonas* isolates. The MIC was also determined for combinations of AGNPs with each tested antibiotic. Determination of MIC employed broth microdilution technique based on the CLSI standard methodology. Different concentrations of AGNPs were prepared, starting from a stock solution containing 170 µg/ml using a serial two-fold dilution method to reach a concentration containing 0.664 µg/ml. A two-fold dilution was conducted for the tested antibiotic, starting with stock solutions containing 1024 µg/ml.

The bacterial suspension was prepared by colony dilution method. Briefly, 10 ml of MHB was inoculated with a loopful of test organisms. The turbidity was diluted to a concentration equivalent to 0.5 McFarland Standard using sterile saline before further dilution 1:100 with fresh (MHB) medium. Aliquots of the final dilution (100 µl) were loaded into the wells of 96 well plates followed by the addition of 100 µL samples of the prepared concentrations of AGNPs or the tested antibiotics. A well containing 100 µL of bacterial suspension in the absence of silver or antibiotics was employed as a positive control. A well containing sterile MHB was used as negative control. The plates were occluded with their lids and incubated at 37 ^o^C for 24 h at the end, MIC was determined as the lowest concentration showing no visible growth [23].

The minimum bactericidal concentration (MBC) was determined in the case of AGNPs. This involved subculturing 10 µL samples from each well on sterile nutrient agar plates. These were incubated aerobically at 37 ^o^C for 24 h. The MBC was recorded as the lowest concentration showing no colony on the agar plate.

The synergistic potential of AGNPs with antibiotics was monitored by determination of the MIC of each antibiotic in the presence of AGNPs which were included in each well at a concentration equivalent to 0.5 MIC of silver on the corresponding bacteria.

### Calculation of fractional inhibitory concentration (FIC) index

For calculating the index of the fractional inhibitory concentration (FICI), MIC values obtained for a given combination were used to evaluate the effects of the combination between each antimicrobial agent and AGNPs according to the formulas defined by Davidson (1989) [24]:

$$FICA\hspace{0.17em}=\hspace{0.17em}MIC A\hspace{0.17em}+\hspace{0.17em}B / MIC A$$

$$FIC B\hspace{0.17em}=\hspace{0.17em}MIC B\hspace{0.17em}+\hspace{0.17em}A / MIC B$$

$$FIC index\hspace{0.17em}=\hspace{0.17em}FICA\hspace{0.17em}+\hspace{0.17em}FICB.$$

The MIC of compound A in the presence of compound B is represented by the MIC A + B value, and vice versa for MIC B + A. Determination of the MIC for the individual components is required for calculating the FIC value for either substance A or B. The obtained FICI values were used to classify the nature of the interaction as: synergistic (FICI ≤ 0.5), additive (0.5 < FICI ≤ 1, indifferent (1 < FICI < 2), and antagonistic (FICI ≥ 2) [25].

### Detection of biofilm formation and effect of AGNPS on established biofilm

The isolates were tested for biofilm formation using the crystal violet assay described by Christensen et al. (1985) [26]. A loopful of the test bacteria was incubated at 37 °C for 24 h in 10 mL of LB broth. The culture was adjusted to 0.5 McFarland, and 200 µL of this bacterial suspension was transferred to the wells of 96 wells with flat-bottom TCPs. A well-containing sterile medium served as a negative control. The plates were incubated aerobically for 48 h at 37 °C. After incubation, the content of each well was removed, and wells were washed three times with phosphate buffer saline (PBS) to remove planktonic cells. Adherent biofilm was fixed with 95% ethanol for 15 min, followed by staining with 100 µL of 1% crystal violet for 10 min. The unbound stain was removed, and the wells were washed with 200 µl of 33% glacial acetic acid, and the plate was air dried. The stained adherent biofilm’s optical densities (OD) were obtained with an ELISA reader at wave-length 570 nm. Each bacterial isolate was tested in triplicates, and the average OD values were calculated for each isolate and negative control. The cut-off value (OD_C_) was established according to be equal to three standard deviations (SD) above the mean OD of the negative control: OD_C_ = average OD of negative control + (3 × SD of negative control), and the tested isolates were categorized according to biofilm formation into: [27]

1. Non biofilm producer OD ≤ OD_C_
2. Weak biofilm producer OD_C_ < OD ≤ 2×OD_C_
3. Moderate biofilm producer 2×OD_C_ < OD ≤ 4×OD_C_
4. Strong biofilm producer 4× OD_C_ < OD

The antibiofilm activity of AGNPs was then researched. Biofilm were allowed to form as described previously in the presence of 0.5 MIC of AGNPs [6]. The percentage of biofilm inhibition was computed using the following equation:

$$\eqalign{& \% \,{\rm{Of}}\,{\rm{biofilm}}\,{\rm{inhibition}} \cr & \,\,\,\,\,\,\,\,\,\, = 1 - {{OD570\,of\,cells\,treated\,with\,AgNPS} \over {OD570\,of\,untreated\,control}} \times 100 \cr}$$

### Effect of silver NPs on the motility of *Pseudomonas* isolates

The effect of AGNPs on motility was determined using plate assay. Motility assay was conducted by using motility plates containing glucose (1%), bactoagar (0.5%), bactopeptone (0.6%), and yeast extract (0.2%) in the absence and in the presence of 0.5 MIC of AGNPs. The plates were inoculated with a sterile toothpick and incubated at 37 °C for 24 h. Motility was assessed by measuring the zone formed by the colonies migration array from the point of inoculation. The diameter of the swarming zone was then measured in millimeters (mm) (Fig. 4) [28].

### Effect of silver NPs on total proteases and gelatinase production

This was investigated according to the well-established method described by (Gupta et al., 2011; Vijayaraghavan and Vincent, 2013) [29, 30]. Briefly, 0.5 mL of overnight cultures (adjusted to 0.5 MacFarland) were inoculated in 5 mL LB broth both in the presence and absence of 0.5 MIC of AGNPs. These were incubated at 37 ^o^C for 18 h at the end of which the cultures were centrifuged for 15 min at 8500 g and then filtered using a 0.45 μm filter. The supernatant was used for testing the protease and gelatinase activities.

Protease activity was tested using skimmed milk agar. The prepared supernatant (100 µl) was added to the wells made in the skimmed milk agar plates. The plates were incubated overnight for 24 h at 37 ^o^C. The clear zones around the wells were measured and were taken as a measure for protease activity.

Gelatinase activity was similarly monitored using (1.5% LB agar supplemented with 3% gelatin). The existence and diameter of the clear zone were taken as a reflection for gelatinase activity.

### Effect of AGNPs on pyocyanin production by *P. Aeruginosa*

Pyocyanin production was quantified as described by Das and Manefield (2012) [31]. Shortly, *Pseudomonas* suspension obtained after overnight culture in LB broth was diluted to 0.5 McFarland. This bacterial suspension (10 µL) was used to inoculate 1 mL of LB broth in the absence and presence of sub-MIC (0.5 MIC) concentration of AGNPs followed by 48 h incubation at 37 ^o^C. Bacterial cells were separated by centrifugation at 10,000 rpm for 10 min to remove cells. The supernatant was used to quantify of pyocyanin by measuring the absorbance at 691 nm by spectrofluorometer [32].

### Real-time PCR

The RT-PCR was performed in the presence of 0.5 MICs of AGNPs. Total RNA was extracted using Purelink^®^ RNA Mini Kit (Thermo SCIENTIFIC, USA) according to the manufacturer’s instructions. A single colony of each tested isolate was grown in LB broth adjusted to 0.5 MacFarland and incubated at 37 ^o^C for 18 h in the presence and absence of 0.5 MIC of AGNPs. Reverse transcription followed by qRT-PCR of QS-regulatory genes *lasR, rhlR, and pqsR* was carried out using QuantiTects Reverse Transcription Kit (Qiagen, USA). Thermo Scientific Maxima SYBR Green/Fluoresce in qPCR Master Mix (2X) is a ready-to-use solution optimized for quantitative real-time PCR and two-step real-time RT-PCR. It contains SYBR^®^ Green I dye and is supplemented with fluorescein passive reference dye. Only templates and primers need to be added. The SYBR Green I intercalating dye allows DNA detection and analysis without sequence-specific probes. dUTP is included in the mix for optional carryover contamination control using uracil-DNA glycosylase (UDG). Each sample was subjected to real-time PCR in duplicate, and the mean values of the duplicates were used for subsequent analysis. Using 2^−∆∆ct^ [33] relative expression of the target gene was estimated as follows:

Control group was applied as calibrator. On the other hand, other dietaries groups were represented as tested groups for both target and reference genes.

Threshold cycler numbers (Ct) of target gene were normalized to reference genes, for tested and control groups according to following equations:

$$\varDelta Ct \left(tested\right)\hspace{0.17em}=\hspace{0.17em}Ct \left(target\,in\,the\,teste\,groups\right) - Ct (ref. in\,test\,group)$$

$$\varDelta Ct \left(calibrator\right)\hspace{0.17em}=\hspace{0.17em}Ct \left(target\,in\,control\right) - Ct (ref. in\,control)$$

∆ Ct of tested genes were normalized to the ∆ Ct of the calibrator as follows:

$$\varDelta \varDelta Ct = \varDelta Ct \left(test\right) - \varDelta Ct \left(calibrator\right)$$

Relative gene expression fold change was estimated as follows:

Fold changes = (2^−∆∆ct^).

### Statistical analysis

All the experiments were performed in triplicates, and the results were expressed as means ± SD. In order to check the significance of each experiment, Student’s t-test was performed using Microsoft Excel. A value of *p* < 0:05 was considered to be statistically significant.

## Results

### Characterization of silver nanoparticles

**Fig. 1:**
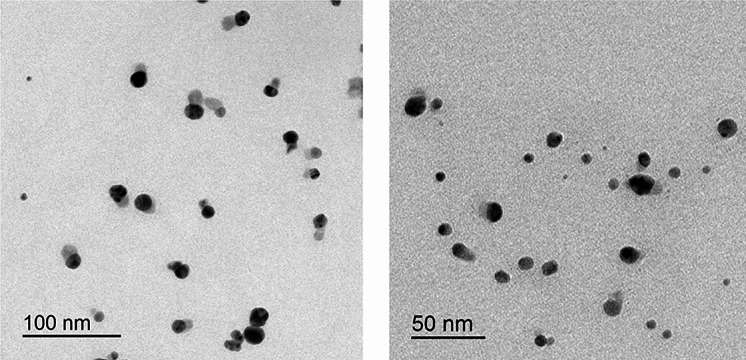
Representative transmission electron micrographs of AGNPs

TEM was used to examine the morphology and the size of AGNPs. The captured micrographs were used to measure the size of AGNPs. Figure 1 shows representative scanning electron micrographs of the prepared AGNPs. The micrographs revealed silver particles which were spherical in shape with an average particle size of 10.84 *±* 4.64 (nm).

**Fig. 2:**
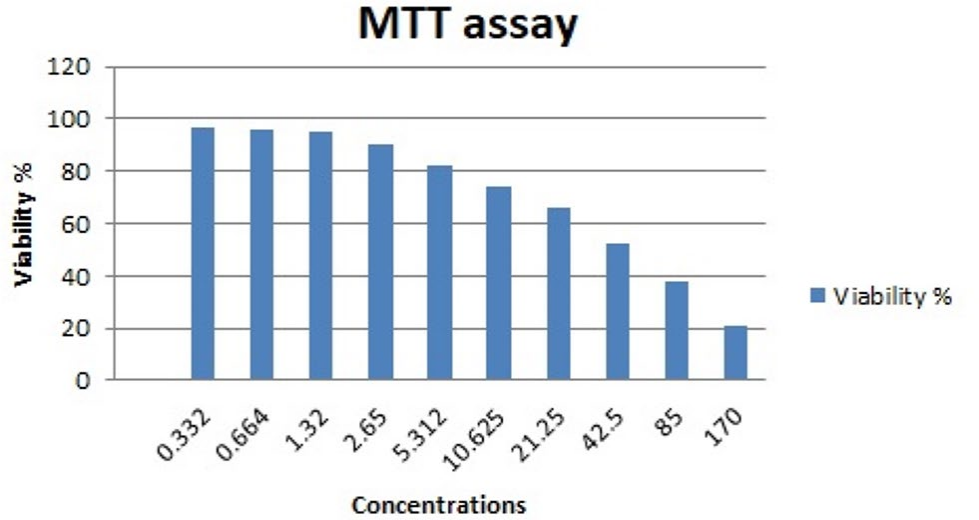
Effect of the concentration µg/ml of AGNPs on the viability of mononuclear cells

The cytotoxic effect of AGNPs was assessed on mononuclear cells. This employed MTT assay. The results are graphically illustrated in Fig. 2. The IC50 was calculated as 42.5 µg/ml.

### Antimicrobial susceptibility testing

**Fig. 3:**
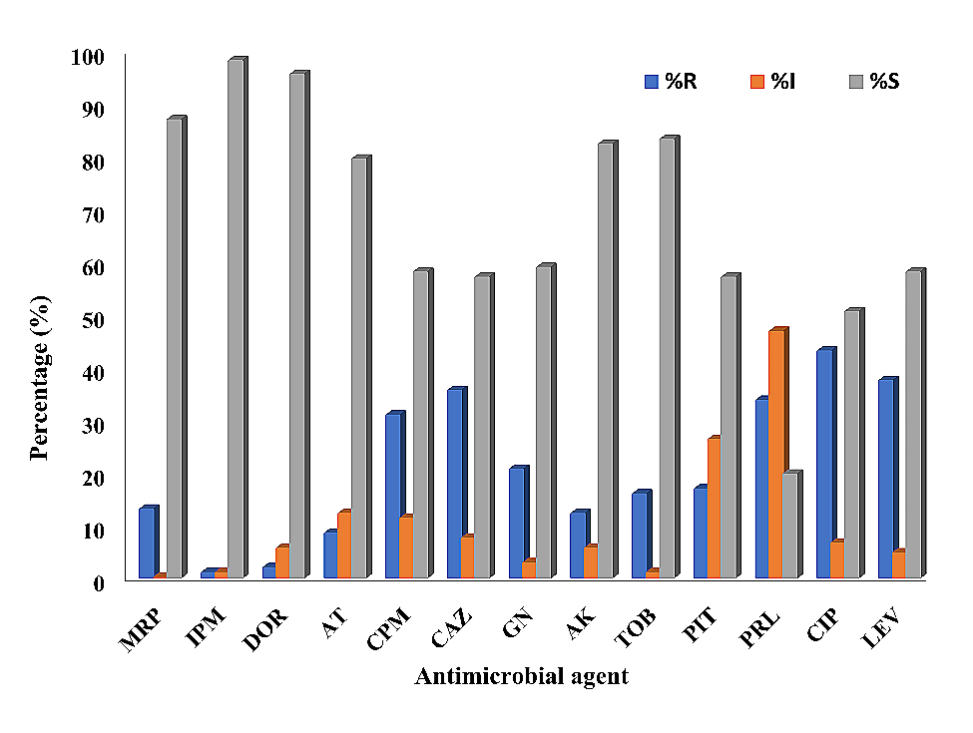
Incidence of resistance of *P. aeruginosa* isolates to different antimicrobials Meropenem 10 µg (MRP), Imipenem 10 µg (IPM), Doripenem 10 µg (DOR), Aztreonam 30 µg (AT), Cefepime 30 µg (CPM), Ceftazidime 30 µg (CAZ), Gentamycin 10 µg (GN), Amikacin 30 µg (AK), Tobramycin 10 µg (TOB), Piperacillin tazobactam 10 µg (PIT), Piperacillin 100 µg (PRL), Ciprofloxacin 5 µg (CIP) and Levofloxacin 5 µg (LEV)

All *P. aeruginosa* isolates (107) were subjected to susceptibility testing against different antimicrobials using the agar dilution. The results were interpreted based on the inhibition zone diameter into resistant (R), intermediate (I) or sensitive (s) isolates according to the clinical breakpoints provided by the Clinical Laboratory Standards Institute CLSI (2018). The results of this interpretation are shown in Fig. 3. The incidence of resistance depended on the type of antibacterial agent tested, with the incidence ranging from 0.93 to 43% of the tested isolates. Ciprofloxacin exhibited the most significant resistance among the tested agents, with 43% of the bacterial isolates categorized as resistant. Levofloxacin was ranked second, with 37.38% of isolates being resistant to it. Ceftazidime was the third in this category, with the incidence of resistance being 35.51%. Imipenem showed the most minor incidence of resistance, with only 0.93% of the isolates showing resistance.

### Antimicrobial resistance patterns of the tested isolates

Resistance patterns and MAR indices of all tested isolates against the studied antimicrobial drugs are presented in Table 2. The results indicated the existence of heterogonous resistance patterns in 63 isolates out of the tested 107. The magnitude of resistance of bacterial isolates was further reflected from the calculated MAR index values, with high values indicating resistance to larger number of antimicrobial agents. The MAR indices ranged from 0.077 to 0.84, with 4 (6%) isolates having a MAR index of 0.84 (Table 2). Based on the recorded resistance pattern, the isolates were classified as MDR, XDR, and PDR. Fortunately, none of the tested isolates exhibited a PDR profile. However, out of 63 *P. aeruginosa* isolates, 12 (19%) and 13 (20.6%) showed MDR and XDR profiles (Table 2). Based on the resistance pattern of the MDR and XDR isolates, ciprofloxacin, ceftazidime, and gentamycin were selected for further investigations.

**Table 2:** Antimicrobial resistance patterns, resistance profiles and MAR indices of *P. Aeruginosa* tested isolates

Antimicrobial resistance pattern			No. of isolates		Isolates exhibiting the pattern	Resist. profile	MAR index
Pattern code		Resistance markers					
Ps1	a	CAZ	8		61-63-65-71-77-80-87-89	----	0.077
	B	PRL	8		30-32-36-37-38-39-40-45		
	c	AK	1		41		
	d	CIP	1		44		
Ps2	a	CAZ-PRL	1		69	----	0.15
	b	CAZ-PIT	1		74		
	c	CIP-LEV	4		11-13-14-67		
	d	AT-PIT	1		29		
Ps3	a	CIP-LEV-PRL	3		5-8-10	----	0.23
	b	CPM-CIP-LEV	5		1-6-7-12-59		
Ps4	a	CPM-CIP-LEV-PRL	1		9	MDR	0.30
	b	CPM-CAZ-CIP-LEV	4		88-78-95-70	----	
	c	GN-CIP-LEV-TOB	1		107		
Ps5	a	CPM-GN-CAZ-PIT-CIP-PRL	1	85		MDR	0.46
	b	MRP-GN-TOB-CAZ-CIP-LEV	1				
					22		
Ps6	a	CPM-CAZ-GN-AK-TOB-CIP-LEV	1		68	MDR	0.54
	b	MRP-AT-CAZ-GN-PIT-CIP-LEV	1		17	XDR	
	c	CPM-CAZ-GN-PIT-CIP-LEV-PRL	1		94	MDR	
	d	CPM-CAZ-GN-TOB-CIP-LEV-PRL	2		25–66	MDR	
	e	MRP-GN-CAZ-PIT-CIP-LEV-PRL	1		102	MDR	
	f	MRP-CPM-CAZ-GN-CIP-LEV-PRL	1		49	MDR	
	g	AT-CPM-CAZ-GN-CIP-LEV-PRL	1		53	MDR	
Ps7	a	CPM-CAZ-GN-AK-PIT-CIP-LEV-PRL	1		79	XDR	0.61
	b	MRP-CPM-CAZ-GN-PIT-CIP-LEV-PRL	1		55	MDR	
	c	CPM-CAZ-GN-TOB-PIT-CIP-LEV-PRL	1		64	MDR	
Ps8	a	AT-CPM-CAZ-PIT-GN-TOB-CIP-LEV-PRL	1		72	XDR	0.69
	b	MRP-CPM-CAZ-GN-AK-TOB-CIP-LEV-PRL	1		86	XDR	
	c	MRP-DOR-CPM-GN-AK-TOB-CIP-LEV-PRL	1		57	XDR	
Ps9	a	MRP-CPM-CAZ-GN-AK-TOB-PIT-CIP-LEV-PRL	2		15–16	XDR	0.77
	b	AT-CPM-CAZ-GN-AK-TOB-PIT-CIP-LEV-PRL	1		76	XDR	
	c	MRP-DOR-IPM-CPM-GN-AK-TOB-CIP-LEV-PRL	1		42	XDR	
Ps10	a	MRP-DOR-CPM-CAZ-GN-AK-TOB-PIT-CIP-LEV-PRL	3		33-34-73	XDR	0.84
	b	MRP-AT-CPM-CAZ-GN-AK-TOB-PIT-CIP-LEV-PRL	1		84	XDR	

### Minimum inhibitory concentration (MIC)

Table 3 shows the minimum inhibitory concentration (MIC) of AGNPs alone and in combination with gentamicin, ceftazidime, and ciprofloxacin against *P. aeruginosa* selected clinical isolates (25). It was found that the MIC values of AGNPs against tested isolates ranged from 2.65 to 21.25 µg/mL, and the MBC values ranged from 10.625 to 42.5 µg/mL (Table 3). The MIC values of the three tested antibiotics were determined in the absence and presence of 0.5 MIC of AGNPs against the bacterial isolates. In the case of gentamicin, MIC values decreased markedly in the presence of AGNPs, with a 4-9-fold reduction in the MIC being recorded. This reduction was manifested by recording MIC values of 1 µg/mL in 92% of the isolates. For ceftazidime, combination with AGNPs resulted in 1-9-fold reduction in the MIC values. The MIC values were reduced to reach as low as 1 µg/mL in 13 (52%) isolates. The combination of AGNPs with ciprofloxacin reduced its MIC by 3-10-fold, with the lowest MIC value (0.5 µg/mL) being recorded in 19 (76%) isolates (Table 3). The reduction in the MIC was further evaluated by calculation of the Fractional Inhibitory Concentration Index (FICI), which was employed to assess the existence of synergistic (FICI ≤ 0.5), additive (0.5 < FICI ≤ 1), indifferent (1 < FICI < 2), or antagonistic (FICI ≥ 2) as represented in Table 4. The results revealed that AGNPs highly synergized the action of aminoglycosides, cephems and fluoroquinolones against *P. aeruginosa;* specifically, gentamicin combined with 0.5 MIC of AGNPs showed synergism against approximately 96% of tested isolates of *P. aeruginosa*. The results also indicated that the combination of ciprofloxacin with 0.5 MIC of AGNPs had a synergistic mode of interaction against 84% of tested isolates. AGNPs provided a synergistic effect for ceftazidime in case of 76% of tested isolates (Table 4).

**Table 3:** Minimum inhibitory concentration (MIC) (µg/ml) of silver nanoparticles (SN), gentamicin (GN), Ceftazidime (CAZ), ciprofloxacin (CIP) and their combination

Isolate No	SN	GN	GN + SN	CAZ	CAZ + SN	CIP	CIP + SN
9	21.25(42.5)	32	1	512	1	32	0.5
15	21.25(42.5)	16	1	128	1	64	0.5
16	21.25(42.5)	512	1	256	1	64	0.5
17	21.25(42.5)	512	1	512	1	64	0.5
22	21.25(42.5)	512	1	128	4	64	0.5
25	21.25(42.5)	16	1	32	4	512	0.5
33	21.25(21.25)	16	1	128	1	256	0.5
34	21.25(42.5)	512	1	512	256	64	0.5
42	10.625(42.5)	512	1	32	16	64	0.5
49	2.656(10.625)	512	1	256	8	512	16
53	5.312(10.625)	16	1	256	128	512	1
55	5.312(21.25)	64	1	128	64	256	0.5
57	10.625(42.5)	256	1	512	256	512	0.5
64	10.625(10.625)	512	1	256	2	128	16
66	2.656(10.625)	512	1	256	1	512	64
68	5.312(10.625)	512	4	512	2	512	64
72	21.25(42.5)	512	2	128	1	256	0.5
73	21.25(42.5)	512	1	256	2	256	0.5
76	21.25(21.25)	512	1	128	2	512	0.5
79	21.25(21.25)	512	1	256	1	512	0.5
84	21.25(21.25)	512	1	256	1	32	0.5
85	10.625(10.625)	512	1	128	1	512	0.5
86	21.25(21.25)	512	1	256	1	128	0.5
94	5.312(21.25)	512	1	64	1	512	0.5
102	10.625(21.25)	64	1	64	1	64	1

*Values between brackets are MBC of silver nanoparticles

**Table 4:** Fractional inhibitory concentration index (FICI) and the effect of combining silver nanoparticles (SN) with three antibiotics (gentamicin (GN), ceftazidime (CAZ) and ciprofloxacin (CIP))

Sample	FICI GN + SN	Effect	FICI CAZ + SN	Effect	FICI CIP + SN	Effect
9	0.078	Synergy	0.049	Synergy	0.038	Synergy
15	0.109	Synergy	0.054	Synergy	0.31	Synergy
16	0.049	Synergy	0.051	Synergy	0.31	Synergy
17	0.049	Synergy	0.049	Synergy	0.31	Synergy
22	0.049	Synergy	0.219	Synergy	0.31	Synergy
25	0.109	Synergy	0.313	Synergy	0.024	Synergy
33	0.109	Synergy	0.054	Synergy	0.025	Synergy
34	0.049	Synergy	12.5	Antagonism	0.030	Synergy
42	0.096	Synergy	2	Additive or indifference	0.054	Synergy
49	0.378	Synergy	3	Additive or indifference	6	Antagonism
53	0.25	Synergy	24.5	Antagonism	0.19	Synergy
55	0.20	Synergy	12.5	Antagonism	0.096	Synergy
57	0.098	Synergy	24.5	Antagonism	0.048	Synergy
64	0.096	Synergy	0.19	Synergy	1.625	Additive or indifference
66	0.378	Synergy	0.37	Synergy	24.125	Antagonism
68	0.760	Additive or indifference	0.37	Synergy	12.125	Antagonism
72	0.098	Synergy	0.054	Synergy	0.025	Synergy
73	0.049	Synergy	0.10	Synergy	0.025	Synergy
76	0.049	Synergy	0.11	Synergy	0.024	Synergy
79	0.049	Synergy	0.051	Synergy	0.024	Synergy
84	0.049	Synergy	0.051	Synergy	0.038	Synergy
85	0.096	Synergy	0.10	Synergy	0.048	Synergy
86	0.049	Synergy	0.051	Synergy	0.027	Synergy
94	0.190	Synergy	0.20	Synergy	0.095	Synergy
102	0.109	synergy	0.11	Synergy	0.11	Synergy

### Antibiofilm activity of silver nanoparticles

The tested isolates were screened for biofilm production using a crystal violet assay. The results of this screening are presented in Table 5. It was found that samples no. 25, 42, 64, and 85 (16%) are strong biofilm producers, isolates no. 9, 15, 16, 17, 22, 33, 34, 49, 57, 72, 79, 84, and 102 (52%) are moderate biofilm producers and isolates no. 53, 55 ,66, 68, 73, 76, 86, and 94 (32%) are weak biofilm producers. The results of 0.5 MIC of AGNPs against *Pseudomonas* biofilm are represented in Table 5. The percentage of biofilm inhibition ranged from 2 to 89% and it was found that AGNPs decreased biofilm formation by more than 50% in about 40% of the tested isolates. Statistical analysis studies revealed that the effect of AGNPs on *Pseudomonas* isolates biofilm is statistically significant (*P* < 0.05) in most of the isolates except isolates no. 33 and 55 only (Table 5).

**Table 5:** Percentage of biofilm inhibition of AGNPs against *P. Aeruginosa* isolates

Sample	Biofilm forming ability	% inhibition
9	Moderate	17 ± 1.026
15	Moderate	35.7 ± 3.30
16	Moderate	24.25 ± 2.10
17	Moderate	48 ± 4.430
22	Moderate	53.37 ± 10.5
25	Strong	58.68 ± 18.9
33	Moderate	2 ± 0.50
34	Moderate	41.6 ± 6.38
42	Strong	42 ± 10.97
49	Moderate	65 ± 7.09
53	Weak	89 ± 29.6
55	Weak	10 ± 1.40
57	Moderate	32 ± 5.63
64	Strong	62 ± 12.45
66	Weak	74.2 ± 2.968
68	Weak	54 ± 2.4
72	Moderate	51.5 ± 14.12
73	Weak	54.4 ± 2.47
76	Weak	47 ± 3.25
79	Moderate	36.8 ± 3.80
84	Moderate	48.4 ± 5.71
85	Strong	76.4 ± 23.96
86	Weak	27.3 ± 2.32
94	Weak	40.6 ± 7.28
102	Moderate	26 ± 2.32

### Effect of silver nanoparticles on the motility of pseudomonas isolates

**Fig. 4:**
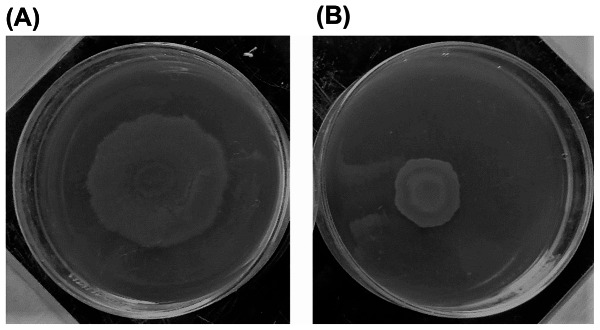
*Pseudomonas* motility in the absence (**A**) and presence (**B**) of AGNPs

The effect of AGNPs on the motility of *Pseudomonas* isolates was investigated by monitoring the swarming of the organism. The diameter of the swarming motility zone was assessed. This is exemplified in Fig. 4. The recorded zone diameters of the tested isolates are presented in Table 6 in the absence and presence of 0.5 MIC of AGNPs. In the absence of AGNPs, bacterial isolates were able to move within the agar plate with the swarming zone diameter ranging from 18 to 37 mm. In the presence of 0.5 MIC of AGNPs, the motility of the organism was reduced, with the swarming zone diameter values being in the range of 10 to 29 mm depending on the tested isolate. The diameter of the zone after treatment of the given isolate with AGNPs was correlated with the corresponding zone diameter in the absence of silver to compute the percentage inhibition of motility. This computation reflected a variable % reduction in motility, with the maximum reduction being 66.6% in isolates 49 and 66 (Table 6).

**Table 6:** Motility zone diameter (mm) in the absence and presence of 0.5 MIC (µg/ml) AGNPs

Sample	control	0.5 MIC AGNPS	% Reduction
9	32	27	15.6
15	33	22	54.5
16	20	15	40
17	25	16	60
22	37	20	59.4
25	20	13	50
33	18	14	22
34	18	10	55.5
42	20	15	25
49	26	20	66.6
53	20	14	55
55	35	25	28.5
57	30	29	46.6
64	30	23	63.3
66	30	25	66.6
68	25	23	52
72	25	20	33.3
73	23	18	34.8
76	25	23	40
79	30	24	20
84	25	20	32
85	27	22	57
86	28	23	17.8
94	35	20	42.5
102	30	23	23

### Effect of silver nanoparticles on the protease and gelatinase production

**Fig. 5:**
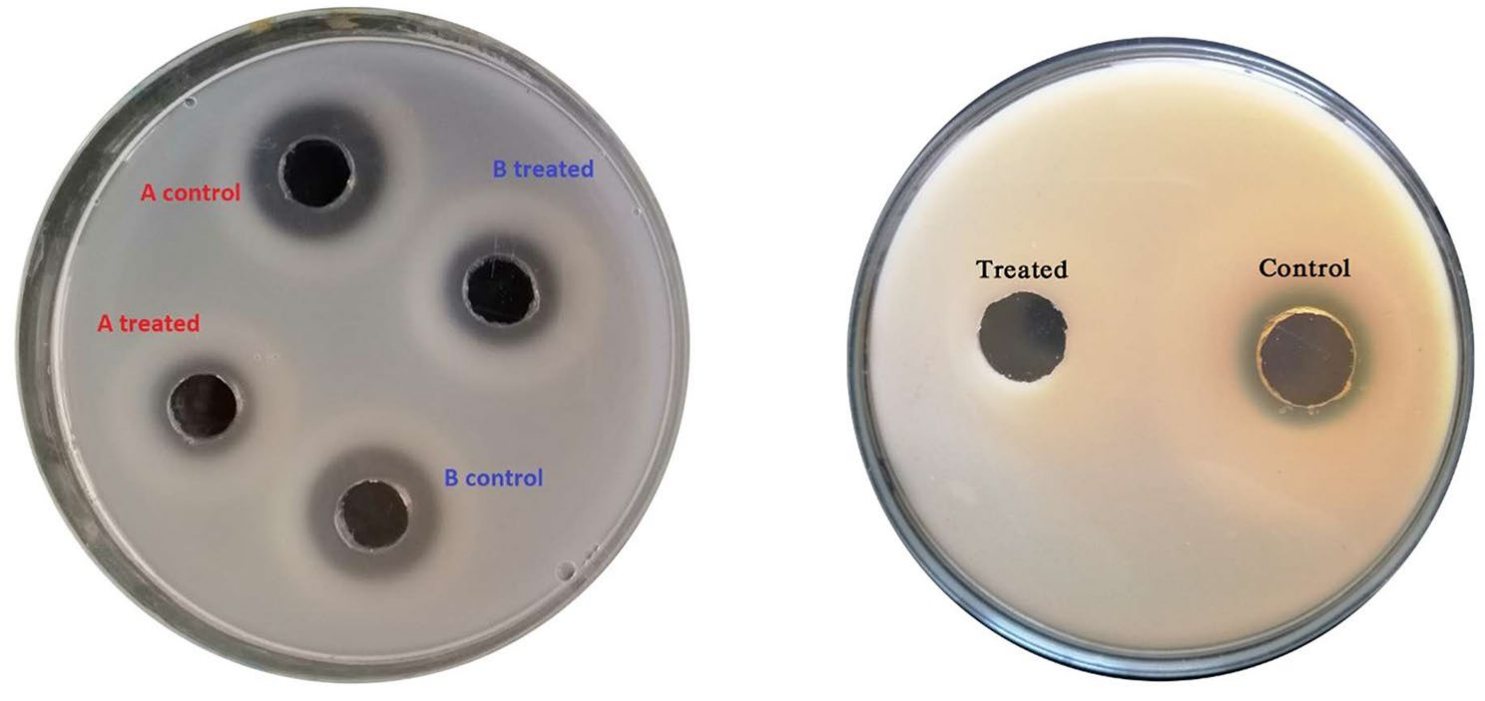
Examples of protease (**left**) and gelatinase (**right**) activities of *Pseudomonas* isolate in absence and presence of 0.5 MIC of AGNPs

Protease and gelatinase assay were conducted in the absence and presence of 0.5 MIC of AGNPs. Proteolytic activity and gelatinase activity was detected on the skimmed milk agar plates and 1.5% LB agar supplemented with 3% gelatin, respectively. The proteolytic and gelatinase activities were shown as clear zones, as exemplified in Fig. 5. The clear zone diameter was used to estimate the effect of AGNPs, as presented in Table 7. The proteolytic activity data reflected the ability of 0.5 MIC concentration of AGNPs to reduce the ability of the tested isolates to secrete protease enzyme with the magnitude of reduction ranging between 5.88 and 52.38%. It should be noted that AGNPs failed to reduce the proteolytic activity in the case of one isolate out of the tested 25 isolates (Table 7). concerning gelatinase activity, a concentration equivalent to 0.5 MIC of AGNPs showed a significant reduction in gelatinase activity with a degree of reduction ranging from 13 to 100% (Table 7).

**Table 7:** Effect of AGNPs on protease and gelatinase activities expressed as the percentage change in activity zone diameters (mm) after treatment with 0.5 MIC (µg/ml) AGNPs

Sample	Protease activity			Gelatinase activity		
	Control	0.5 MIC AGNPS	% Reduction	Control	0.5 MIC AGNPS	% Reduction
9	19	15	21	20	15	25
15	19	14	26.3	19	12	36.84
16	17	15	11.76	15	13	13.3
17	19	14	26.3	19	12	36.8
22	20	13	35	16	11	31.25
25	21	15	28.6	20	12	40
33	17	16	5.88	15	13	13.3
34	20	15	25	17	13	23.5
42	20	15	25	19	14	26.3
49	21	14	33.33	20	11	45
53	15	9	40	15	00	100
55	14	14	00	12	11	8.33
57	19	15	21	17	13	23.5
64	22	11	50	20	9	55
66	15	9	40	13	8	38.5
68	15	11	26.6	15	10	33.33
72	20	16	20	18	11	39
73	15	11	26.66	12	9	25
76	15	11	26.66	15	11	26.6
79	20	14	30	20	12	40
84	19	12	36.8	17	11	35.3
85	21	10	52.38	18	00	100
86	14	12	14.28	15	13	13.3
94	15	12	20	16	12	25
102	20	16	20	20	15	25

### Effect of silver nanoparticles on the pyocyanin production

**Fig. 6:**
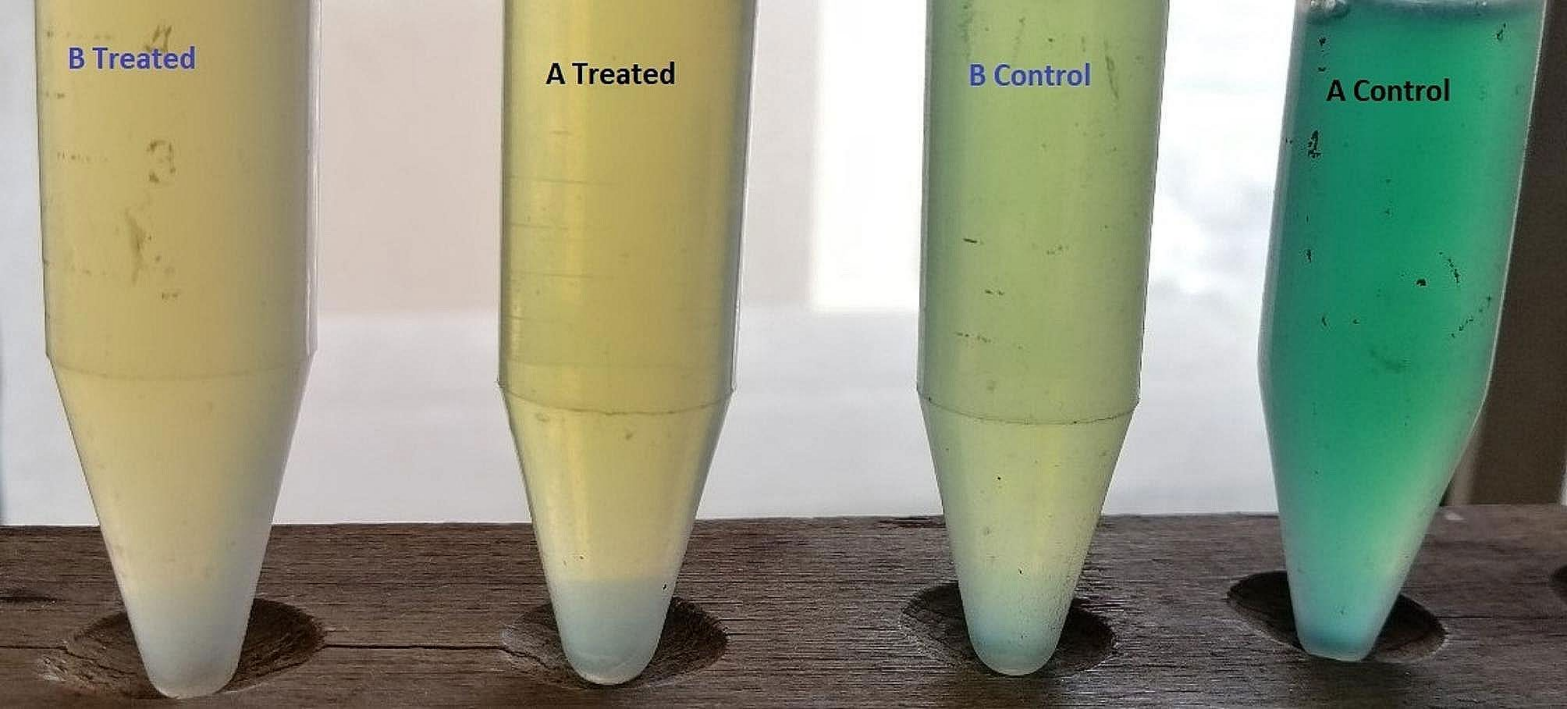
Reduced pyocyanin color after treatment with 0.5 MIC of AGNPs

The effect 0.5 MIC of AGNPs on the ability of *P. aeruginosa* to produce pyocyanin was investigated. The existence of pyocyanin is indicated by the green coloration of the solution with the color fading by inhibition of pyocyanin production (Fig. 6). The OD691 of *P. aeruginosa* pyocyanin after extraction in the absence and presence of 0.5 MIC of AGNPs is represented in Table 8 and was used for quantitative determination. The results showed that pyocyanin concentration decreased after adding AGNPs, with the magnitude of reduction ranging from 9 to 79%, depending on the tested isolate (Table 8).

**Table 8:** Pyocyanin absorbance (nm) in absence and presence of 0.5 MIC (µg/ml) of AGNPs

Sample	Control	0.5 MIC AGNPS	% Reduction
9	0.5	0.37	26
15	0.56	0.32	42.8
16	0.61	0.45	25.8
17	0.66	0.35	47
22	0.56	0.25	55.3
25	0.59	0.24	59.3
33	0.44	0.40	9
34	0.48	0.25	48
42	0.58	0.33	43
49	0.55	0.13	76.3
53	0.35	0.10	71.4
55	0.31	0.26	16
57	0.59	0.36	39
64	0.74	0.27	63.5
66	0.32	0.08	75
68	0.77	0.29	62.3
72	0.52	0.24	54
73	0.36	0.17	53
76	0.43	0.24	44
79	0.52	0.31	40.3
84	0.67	0.33	50.7
85	0.81	0.17	79
86	0.39	0.25	36
94	0.42	0.23	45
102	0.5	0.34	33.3

### Real-time PCR results

**Fig. 7:**
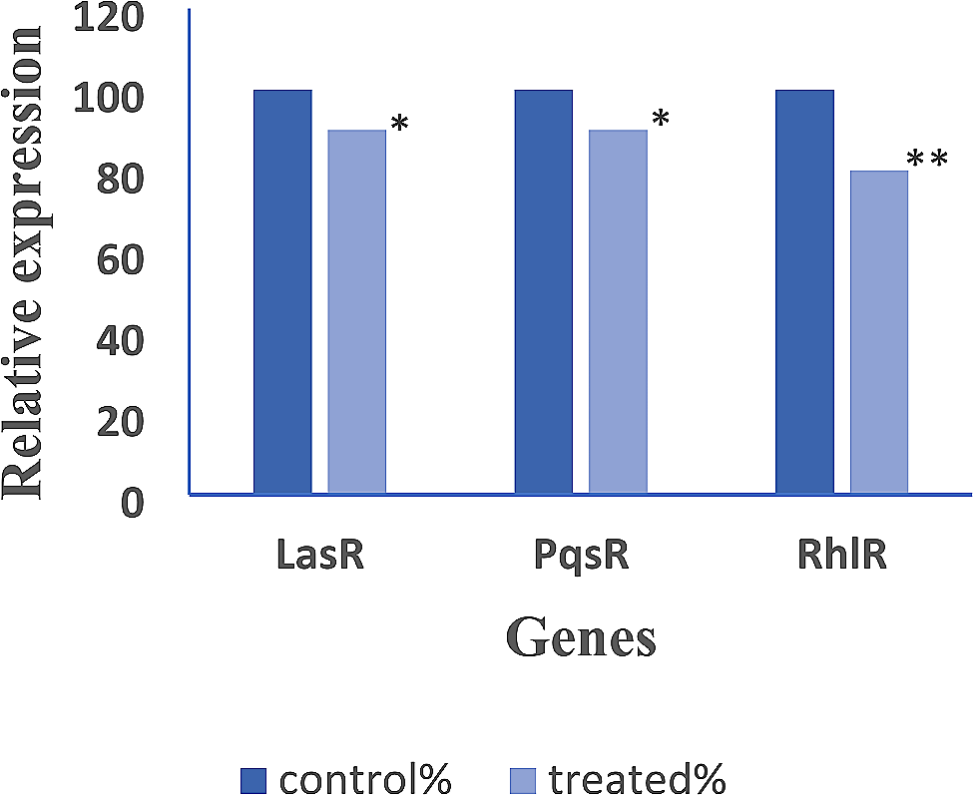
Reduction in the expression of Quorum sensing genes in *P. aeruginosa* isolates treated with AGNPs. Statistical analysis reflected significant reduction after treatment with AGNPs and represented in the charts as * where * means that (*p* < 0.05) and ** means that (*p* < 0.01)

The relative expression of the genes regulating virulence factors production was assessed in AGNPs treated and untreated strains and analyzed using the 2^-∆∆Ct^ method. The relative expression levels of *LasR, PqsR, and RhlR* were significantly reduced under AGNPs sub-MIC treatment, as presented in Fig. 7. The relative expression of *LasR* gene was significantly decreased from 100 to 90%, 80%, 50%, and 40% in isolates no. 9, 42, 64, and 85 after treatment with AGNPs, respectively. Moreover, the relative expression of *PqsR* gene was significantly reduced to 90%, 70%, 60%, and 50% in isolates no. 9, 42, 64, and 85 respectively after AGNPs treatment. Furthermore, the relative expression of *RhlR* was significantly diminished to 80%, 80%, 60%, and 40% in isolates no. 9, 42, 64, and 85 AGNPS treated isolates, respectively (Fig. 7). The statistical analysis studies showed statistically significant difference (*P* < 0.05) in the gene expression after treatment with AGNPs.

## Discussion

Overuse and misuse of antibiotics resulted in the development of resistance to most antibiotics. This requires the development of new drug candidates, a process that is time-consuming and very expensive. The advancement of nanotechnology highlighted the possibility of enhancing the activity of existing antibiotics. Metallic nanoparticles showed promising data, with AGNPs being widely tested [19]. However, the mechanism of action of AGNPs was not systematically researched. The objective of this study was to probe the anti-*Pseudomonas* activity of AGNPs. The synergistic effect on the antibacterial activity of selected antibiotics was also tested. The goal was extended to probe the mechanism of enhanced antibacterial activity by AGNPs. AGNPs were prepared and the morphology and size of the developed particles correlated with the published data on AGNPs prepared by the same technique [34].

Cytotoxicity studies confirmed the safety of AGNPs as revealed from high IC50 values which correlates with the values reported by other investigators. AGNPs (25 nm) were shown to cause death of the human fibroblasts at concentrations above 60 mg/L with silver ions inducing cell death at much lower concentration (2 mg/L). This study reflects the safety of AGNPs and supports our findings [35].

The susceptibility testing of *Pseudomonas* isolates for the tested antibiotics showed a high incidence of resistance against ciprofloxacin, followed by levofloxacin, then ceftazidime with imipenem showing the least incidence of resistance. This rank is comparable with that reported by other investigators who classified amikacin as the most active drug against *P. aeruginosa* followed by meropenem, cefepime, and fluoroquinolones [36]. P. aeruginosa was resistant to ampicillin, amoxicillin and their combination with B-lactamase inhibitors. These data were recovered after testing various antibiotics against *Pseudomonas* isolates collected from Egyptian hospitals [36].

The recorded MAR values and the estimated MDR and XDR profiles reflect the frequent use of the tested antibiotics, which contributed to the development of resistance [37, 38]. Based on the resistance pattern of the MDR and XDR isolates, ciprofloxacin, ceftazidime, and gentamycin were selected for further investigations.

The recorded MIC and MBC values of AGNPs against the tested isolates indicated the anti-*Pseudomonas* activity of AGNPs. The efficacy of AGNPs against *P. aeruginosa* was reported in other studies which monitor this activity by assessing the inhibition zone recording 17 mm in the case of *P. aeruginosa* [34]. Likewise, AGNPs were effective against other bacteria including *Escherichia coli, Enterococcus faecalis, Klebsiella pneumoniae, Acinetobacter baumannii, Listeria monocytogenes, and Micrococcus luteus* [8]. Combination of AGNPs with gentamicin, ceftazidime or ciprofloxacin enhanced their activity as revealed with reduction of MIC compared with the use of net antibiotic. The Fractional Inhibitory Concentration Index (FICI) reflected high potential for synergism. The potentiation of the antimicrobial activity of drugs after combination with AGNPs has been shown by other investigators in case of antibiotics such as neomycin, gentamycin, ciprofloxacin, vancomycin and trimethoprim. The recorded activity of the combinations was better than that of the corresponding antibiotic alone [19, 38].

The effect of AGNPs on *P. aeruginosa* isolates virulence factors associated with its pathogenicity was researched to elucidate the mechanism of enhanced antibacterial activity by AGNPs. This was achieved by monitoring their effect on biofilm, swarming motility, the activity of protease, and gelatinase enzyme and the effect on pyocyanin production in addition to, the effect of AGNPs on the expression of genes encoding various virulence factors.

The ability of *P. aeruginosa* to develop biofilm contributes to its widespread infections and decrease the effectiveness of antibiotics resulting in extended duration of therapy or wrong use of antibiotics which leads to development of microbial resistance [39]. The antibiofilm activity of AGNPs was shown irrespective to the degree of biofilm formation. The antibiofilm activity has been documented by other investigators. The studies recorded antibiofilm efficacy of AGNPs against *Pseudomonas*, but the authors employed two concentrations of AGNPs (50 and 100 nM) without predetermination of the MIC of AGNPs. The results of this investigation reflected concentration dependent antibiofilm activity for AGNPs with 100 nM producing 95% reduction in biofilm activity with 50% reduction being shown with 50 nM [40]. These results support our finding which employed 0.5 MIC of AGNPs. The recoded effect in case of MIC implies that the antibiofilm activity is one of the principal mechanisms for AGNPs induced augmentation of efficacy of other antibacterial agents. The antibiofilm activity of AGNPs was also shown against *Klebsiella pneumoniae* after incubation with AGNPs at concentrations above 75 µg/ml as well suggesting possibility for wider application of AGNPs [41].

The effect of AGNPs on the motility of *Pseudomonas* isolates was investigated by monitoring swarming of the organism. The presence of 0.5 MIC of AGNPs reduced the motility of bacteria. Taking the fact that swarming is involved in development of bacterial resistance with swarming cells showing greater ability to express the genes associated with production of virulence factors, reduction of swarming can be considered a reflection for reduction of resistance [42]. Accordingly, the current data highlight the potential of AGNPs to reduce the ability of *P. aeruginosa* to develop resistance against antibiotics. Combination of AGNPs with an antibacterial can provide the benefit of the antimicrobial activity of AGNPs, in addition to possible reduction in bacterial resistance to the given antibacterial. Many compounds have been shown to reduce the swarming motility of *P. aeruginosa*. These included drugs such as paracetamol, and chemical compounds, such as cinnamaldehyde, alginate oligomer (OligoG), 1-naphthol and other bicyclic compounds bearing hydroxyl groups [32, 042–44].

Protease and gelatinase production were reduced in presence of 0.5 MIC of AGNPs. Considering that these enzymes help bacteria in the process of invasion and colonization, the ability of AGNPs to reduce their production can be considered an evidence for the high potential of AGNPs to reduce the ability of *Pseudomonas* to develop resistance. Many compounds have been shown to reduce the protease and gelatinase activities of *Pseudomonas* isolates. These included drugs, such as paracetamol, azithromycin, erythromycin and clarithromycin and chemicals like Zinc peroxide nanoparticles (ZnO2 -NPs) [32, 45, 46].

AGNPs reduced the ability of *P. aeruginosa* to produce pyocyanin. As for other factors, the ability of AGNPs to reduce the ability of bacteria to produce pyocyanin further supports the potential of AGNPs to reduce development of bacterial resistance. Pyocyanin is believed to play a role in biofilm formation. The existence of pyocyanin in the lungs at high concentration was able to negatively influence the immune response and impair epithelial cell function in patients with cystic fibrosis. Therefore, reduction of pyocyanin production can be considered a promising tool in the therapeutic effect of AGNPs in *P. aeruginosa* infections. The inhibitory effect of pyocyanin production by AGNPs was shown by other investigators [47]. Paracetamol also showed a potential to reduce pyocyanin production by *P. aeruginosa* [32].

Noteworthy, AGNPs can exhibit antibacterial activity by production of reactive oxygen species (ROS) which can induce lipid peroxidation, oxidative protein carbonylation, and inactivation of specific enzymes. This was shown as bactericidal activity against MDR *Klebsiella pneumonia* after conjugating fungal glucan isolated from *Pleurotus florida* with AGNPs [48].

Mechanistic investigations were fortified by real-time PCR to monitor the effect of AGNPs on expression of genes responsible for regulation of virulence factors. The study revealed high potential to reduce the expression of these genes after exposure to AGNPs. The recorded effect is similar to that shown for other metallic nanoparticles like zinc oxide [7]. The PCR data confirm the recorded effects of AGNPs on the tested virulence factors. Overall, AGNPs can augment the effect of antibiotics against *P. aeruginosa* by combined mechanisms including inhibition of biofilm formation and reduction of the activity of virulence factors. The later may take place by interference with the expression of genes responsible for production of these virulence factors.

## Conclusion

AGNPs were successfully prepared with size of 10.84 nm. These nanoparticles were active against *P. aeruginosa* isolates. AGNPs were able to augment the efficacy of ceftazidime, gentamicin or ciprofloxacin against MDR, XDR *P. aeruginosa* isolates with a maximum of 10-fold reduction being achieved in some strains. The efficacy of AGNPs can be attributed to their antimicrobial activity including antibiofilm formation, reduced swarming motility, protease, gelatinase and pyocyanin production in *Pseudomonas* isolates. The study suggests combination of AGNPs with antibiotics for effective treatment of MDR, XDR *P. aeruginosa* infections. Further studies are required to assess the effect of AGNPs in combination with antibiotics systematically (in vivo).

## Data Availability

All data generated or analyzed during this study are included in this published article and raw data will be available upon request.
